# The role of pannexin hemichannels in inflammation and regeneration

**DOI:** 10.3389/fphys.2014.00063

**Published:** 2014-02-25

**Authors:** Helen P. Makarenkova, Valery I. Shestopalov

**Affiliations:** ^1^Department of Cell and Molecular Biology, The Scripps Research InstituteLa Jolla, CA, USA; ^2^Department of Ophthalmology, Bascom Palmer Eye Institute, University of Miami School of MedicineMiami, FL, USA; ^3^Department of Cell Biology and Anatomy, Vavilov Institute for General GeneticsMoscow, Russia

**Keywords:** pannexin, connexin, Panx1, inflammation, regeneration, stem cell, P2X7R, FGF

## Abstract

Tissue injury involves coordinated systemic responses including inflammatory response, targeted cell migration, cell-cell communication, stem cell activation and proliferation, and tissue inflammation and regeneration. The inflammatory response is an important prerequisite for regeneration. Multiple studies suggest that extensive cell-cell communication during tissue regeneration is coordinated by purinergic signaling via extracellular adenosine triphosphate (ATP). Most recent data indicates that ATP release for such communication is mediated by hemichannels formed by connexins and pannexins. The Pannexin family consists of three vertebrate proteins (Panx 1, 2, and 3) that have low sequence homology with other gap junction proteins and were shown to form predominantly non-junctional plasma membrane hemichannels. Pannexin-1 (Panx1) channels function as an integral component of the P2X/P2Y purinergic signaling pathway and is arguably the major contributor to pathophysiological ATP release. Panx1 is expressed in many tissues, with highest levels detected in developing brain, retina and skeletal muscles. Panx1 channel expression and activity is reported to increase significantly following injury/inflammation and during regeneration and differentiation. Recent studies also report that pharmacological blockade of the Panx1 channel or genetic ablation of the Panx1 gene cause significant disruption of progenitor cell migration, proliferation, and tissue regeneration. These findings suggest that pannexins play important roles in activation of both post-injury inflammatory response and the subsequent process of tissue regeneration. Due to wide expression in multiple tissues and involvement in diverse signaling pathways, pannexins and connexins are currently being considered as therapeutic targets for traumatic brain or spinal cord injuries, ischemic stroke and cancer. The precise role of pannexins and connexins in the balance between tissue inflammation and regeneration needs to be further understood.

The vertebrate pannexin family consists of three proteins (pannexin 1, 2, and 3) that have moderate sequence homology with invertebrate innexins and a very low homology with true gap junction proteins, connexins (Panchin et al., 2000; Baranova et al., 2004; Panchin, 2005; D'Hondt et al., 2010). Pannexins and connexins share similar protein structure with four transmembrane domains, two extracellular loops and cytoplasmic tail (Panchin et al., 2000; Baranova et al., 2004; Barbe et al., 2006). In most tissues and cell types, pannexins were shown to form predominantly hemichannels that mediate regulated exchange of second messenger molecules, such as adenosine triphosphate (ATP), between cytoplasm and the extracellular space. Some connexin hemichannels, particularly those formed by connexin isoforms 26, 32, 37, and 43 were also shown to pass ATP molecules in response to phosphorylation, intracellular and extracellular calcium change and other stimuli (Zhao et al., 2005; De Vuyst et al., 2006; Scemes et al., 2007; Shestopalov and Panchin, 2008; Silverman et al., 2008; Sonntag et al., 2009; Bao et al., 2012). However, the two families of channel proteins have quite distinct physiological properties, particularly the ability to form full vs. partial channels *in vivo* and in the spectrum of binding partners (Scemes et al., 2007; Shestopalov and Panchin, 2008; Silverman et al., 2008; Bao et al., 2012). In contrast to connexins, pannexins are insensitive to physiological levels of extracellular calcium, possess faster kinetics of pore opening, larger unitary conductance and very weak voltage gating (Bruzzone et al., 2003; Bao et al., 2004). Pannexin channels actively interact and are regulated/ modulated by P2Y/P2X purinergic, A1/A2 adrenegric, TRPV, and NMDA receptors and are, thus, implicated in cell signaling cascades downstream of these surface receptors. In addition, Panx1 was shown to be sensitive to mechanical stimuli and modulated by the Kvβ3 potassium channels (Bunse et al., 2009), the feature that makes them responsive to mechanical impacts and high extracellular K^+^ observed in many CNS injuries. Sensitivity of pannexins to mechanical stimuli and wide spectrum of interaction with surface molecules allows the cell to employ pannexin channels as the major conduit for ATP release in response to a variety of physiological and pathological stimuli. Although the function of pannexins as the ATP release channels for purinergic/adenosine signaling and propagation of long-distance intercellular Ca^2+^ waves in astrocyte networks is well understood (Bao et al., 2004; Locovei et al., 2006; Suadicani et al., 2012), their role in the processes of inflammation and regeneration has just started to be evaluated. Pannexins, particularly Panx 1 and Panx2, are highly expressed in developing brain, retina, skeletal and cardiac muscles, and glandular tissues (Vogt et al., 2005; Li et al., 2011b; Cea et al., 2012; Kar et al., 2012; Giaume et al., 2013). In this review, we focus on a role pannexins play in inflammation induced regeneration in various tissues.

## Role of pannexins in inflammation and cell death

**Figure 1:**
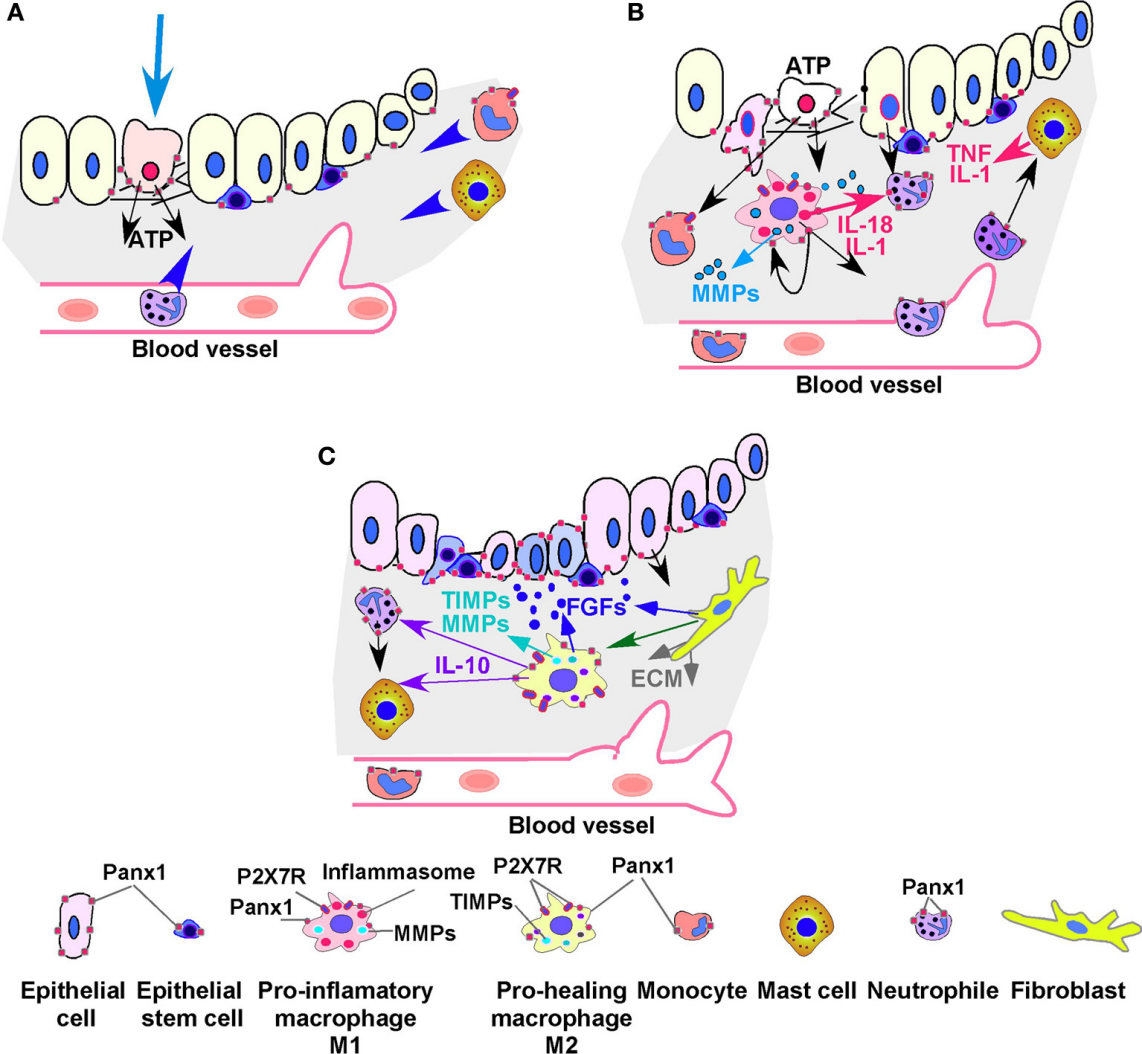
**Scheme of inflammation and wound healing in epithelial tissue**. **(A)** The glandular epithelial cells form a selective permeability barrier separating luminal content from underlying tissues. **(A)** In the epithelial tissue cell damage induces ATP externalization from injured and necrotic cells and later from infiltrating neutrophils, monocytes (macrophages precursors), and macrophages. ATP is released through pannexin hemichannels, specifically through Panx1. ATP activates monocytes, macrophages, and mast cells (also known as effector cells in allergic and inflammatory diseases) that migrate to the site of injury. **(B)** In addition during an inflammatory response, eosinophils and neutrophils migrate from the bloodstream into tissues. They contribute to the recruitment of monocytes and macrophages. Macrophages and mast cells express P2X7 receptors (P2X7Rs), members of the family of ionotropic ATP-gated receptors. ATP activated P2X7Rs are associated with injury activation of the inflammasome within macrophages and transformation of macrophages into pro-inflammatory M1 type of macrophages. M1 macrophages inhibit cell proliferation and induce inflammation by releasing IL-1 and IL-18 and ATP. They also secrete degradative enzymes, such as matrix metalloproteinases (MMPs), collagenase, and elastase, and are crucial in induction of extracellular matrix (ECM) remodeling and tissue reorganization, allowing them and other cells (including epithelial and epithelial stem/progenitor cells) to migrate through tissues. High concentration of ATP can also act as a Panx1 channel inhibitor and thus Panx1 acts as a regulator of its own function (black looped arrow) **(C)** Macrophages are very plastic cells that function as control switches of the immune system, providing a balance between pro- and anti-inflammatory responses. Macrophages could be transformed into pro-healing M2 macrophages by signals released from fibroblasts or by other external signals. M2 macrophages express IL-10, growth factors, and tissue inhibitor of metalloproteinases (TIMPs) suppress immune and inflammatory responses and promote cell proliferation and tissue repair, and angiogenesis. Migrating fibroblasts secrete FGFs and other growth factors that support tissue morphogenesis. Stem and progenitor cells also express pannexin hemichannels, which are involved in regulation of progenitor cell migration and differentiation.

In vertebrates, inflammation often accompanies injury and disease. The immune response to injury is described as an accumulation of both resident (macrophages) and circulating immune cells in the injured tissue. In this process, ATP externalization is the first step in the cascade of events leading to maturation and secretion of inflammatory molecules: interleukin-1α (IL-1α), interleukin-1β (IL-1β), and interleukin-18 (IL-18). In healthy tissues, the amount of ATP released from cells is tightly regulated and its concentration is kept low by extracellular ATP/ADPases (Riteau et al., 2010). In damaged tissues, ATP is released from injured and necrotic cells and from infiltrating monocytes and macrophages through pannexin hemichannels, specifically Panx1 (Figures 1A,B, black arrows). ATP further activates monocytes, macrophages and mast cells (also known as effector cells in allergic and inflammatory diseases) that migrate to the site of injury (Kurashima et al., 2012). Macrophages are very plastic cells that function as control switches of the immune system, providing a balance between pro- and anti-inflammatory responses. In the M1 form, macrophages inhibit cell proliferation and induce inflammation mainly by expressing IL-1 (α and β) and IL-18 (Figure 1B, red arrows) and releasing ATP. M2 macrophages produce anti-inflammatory cytokines and are typically involved in tissue repair (Mills, 2013) (also see below).

Involvement of purinergic receptors in the processes of inflammation and cell death has been well documented (Lister et al., 2007; Burnstock, 2008; Iglesias et al., 2009; Hill et al., 2010; McGilligan et al., 2013). Purinergic receptors function as a ligand-gated ion channels and are responsible for ATP-dependent signaling. The ATP-gated P2X7 purinergic receptors (P2X7Rs) are associated with injury-induced activation of the inflammasome within macrophages (Figure 1B). The inflammasome is a multiprotein complex consisting of caspase-1 and other enzymes that promote the maturation of inflammatory cytokines, such as IL-1β, and IL-18. Initially binding of ATP to P27XR causes opening of a non-selective cation channel; subsequently, with prolonged ATP exposure, a stable non-junctional hemichannel (pore) is formed. Given that a substantial body of evidence demonstrates that Panx1 mediates ATP release in response to stimulation of purinergic receptors in many tissues, including brain and muscles (Brennan et al., 1990; Bruzzone et al., 2003; Baranova et al., 2004; Locovei et al., 2007; Iglesias et al., 2009), Panx1 is believed to form the pore in response to activation of P2X1, P2X4 and P2X7 (Burnstock, 2008; Shestopalov and Panchin, 2008; Feranchak et al., 2010; Woehrle et al., 2010; Lohman et al., 2012; Romanov et al., 2012; Hung et al., 2013). Thus Panx1 mediated ATP release leads to autocrine/paracrine stimulation of P2X7Rs and promotes proinflammatory cytokine processing (Dubyak, 2002; Feranchak et al., 2010; Lohman et al., 2012; Romanov et al., 2012). Using P2X7R^(−/−)^ mice, Pelegrin and coauthors (Pelegrin et al., 2008) provided evidence that activation of P2X7R by ATP in peritoneal macrophages induces inflammasome activation and facilitates the release of IL-1α, IL-18, as well as IL-1β.

Other studies showing roles for both P2X7R and Panx-1 in the control of activation and release of mature IL-1α, IL-1β and IL-18, support the idea that the P2X7Rs/Panx1 signaling complex is a key regulator of inflammatory response (Pelegrin, 2008; Pelegrin et al., 2008; Brough et al., 2009). Intriguingly however, in bone marrow-derived Panx1^−/−^ macrophages IL-1beta and IL-18 were still normally secreted after stimulation with ATP (Qu et al., 2011), suggesting redundancy in the pathway for interleukin activation. At the same time, Panx1^−/−^ thymocytes failed to recruit wild-type peritoneal macrophages, which is consistent with a novel and non-redundant role for Panx1 in releasing nucleotide/find-me signal from apoptotic cells to recruit macrophages (Chekeni et al., 2010). Panx1 also contributes significantly to plasma membrane permeability during apoptosis, which is relevant for “selective” dye uptake by early apoptotic cells (Zhang et al., 2008; Chekeni et al., 2010). Recently, using experimental colitis model, Gulbransen et al. (2012) reported that inflammation causes enteric neuron death by activating a neuronal signaling complex composed of P2X7Rs, Panx1 channels, the ASC adaptor protein and caspases 1 or 11. In this model, as well as in the model of traumatic brain injury (Adamczak et al., 2012), inhibition of either P2X7R, Panx1, ASC or caspase activity prevented inflammation-induced neuron cell death, further supporting an idea that Panx1 mediated signaling pathway is a crucial regulator of cell death (de Rivero Vaccari et al., 2009; Gulbransen et al., 2012).

Panx1 has been also implicated in pathophysiology of many diseases such as Crohn's disease, AIDS, melanoma, epilepsy, chronic intestinal inflammation, spinal cord injury, and stroke (Garre et al., 2010; Kim and Kang, 2011; Santiago et al., 2011; Cowan et al., 2012; Penuela et al., 2012b; Diezmos et al., 2013; Orellana et al., 2013; Oviedo-Orta et al., 2013; Wang et al., 2013). The diversity of the cell states in this diseases suggests that Panx1 function in cellular processes leading to chronic inflammation, cell death, and disease that remain to be fully defined. Moreover, Panx1 signaling most likely provides a positive feedback loop for inflammatory responses involved in acute and chronic inflammation and inflammation related diseases. Manipulation of Panx1 signaling and/or ATP release could be beneficial in treating neutrophil– or T cell-mediated inflammatory diseases.

## Role of pannexins in cell differentiation and tissue regeneration

Several publications suggest that inflammation and regeneration processes are connected and that inflammation is an important prerequisite for regeneration (Filbin, 2006; Godwin et al., 2013; Leibinger et al., 2013; Panayidou and Apidianakis, 2013). The inflammation that accompanies injury and disease can lead to further damage but can also support tissue repair (Wyss-Coray and Mucke, 2002). Tissue regeneration requires the activity of multiple signaling pathways, leading to a blockade of apoptosis, cell proliferation and differentiation and extracellular matrix (ECM) remodeling. Recent reports suggest that inflammation may influence the initiation and completion of wound healing and regeneration (Fahmy and Sicard, 2002; Godwin and Brockes, 2006; Godwin et al., 2013) by modulating and growth factor cytokine microenvironment.

Pannexin mediated ATP release initiates multiple signaling cascades leading initially to recruitment of macrophages, proinflammatory cytokine release and ECM remodeling, and later to growth factor secretion followed by cell migration, proliferation, and differentiation. Especially important is recruitment of macrophages to regenerating tissue (Kharraz et al., 2013; Kim et al., 2013). Recently, immunological signaling was shown to be necessary for limb regeneration in salamanders. Systemic macrophage depletion during salamander limb regeneration resulted in wound closure accompanied by extensive fibrosis, deregulation of ECM components, and complete failure of epimorphic limb regeneration (Godwin et al., 2013). This finding suggests that macrophages produce key regulatory molecules important for regeneration of damaged body parts.

As mentioned above, macrophages can express two major phenotypes depending on the external signals they receive (Mokarram et al., 2012; Mills, 2013). During wound healing, pro-inflammatory M1 macrophages (see above, Figure 1B) are transformed into M2 macrophages that suppress immune and inflammatory responses and promote cell proliferation, tissue repair, and angiogenesis (Mills, 2013) (Figure 1C). M2 macrophages also express anti-inflammatory cytokines, such as IL-10, growth factors, and tissue inhibitor of metalloproteinases (TIMPs). Changes in microenvironmental signals could induce the switch of phenotype from M1 to M2 and vice versa. For example treatment of the regenerating peripheral nerves with IL-4 induced conversion of pro-inflammatory M1 macrophages toward pro-healing M2 macrophages (Mokarram et al., 2012). It has been reported that local fibroblasts that secrete ECM components and participate in ECM remodeling, can also drive macrophages toward the M2-like phenotype (Figure 1C, green arrow) (Comito et al., 2013). In addition M2 macrophages release fibroblast growth factors (FGFs, Figure 1C, blue arrow), that promote epithelial morphogenesis (Figure 1C) (Franzen et al., 1998; Chernykh et al., 2010).

The emerging model is that during the normal process of regeneration, inflammation precedes regeneration, and enhances tissue repair through multiple processes that include cytokines and growth factors release and induction of stem cells proliferation (Martin and Feng, 2009; Kyritsis et al., 2012). For example, release of pro-inflammatory IL-1 causes dose-related secretion of growth factors and pro-healing cytokines that initiate regeneration process (Guenard et al., 1991; Miyauchi et al., 1997). More recent study shows that inflammation is required and sufficient to enhance the proliferation of neural progenitors and neurogenesis in Zebrafish brain (Kyritsis et al., 2012). However, uncontrolled release of IL-1 and other proinflammatory molecules may lead to chronic inflammatory diseases, thus tight temporal and spatial control of these factors must be maintained.

Among the proteins regulating IL-1 processing and release, P2X7R and Panx1 play the role (Qu et al., 2007; Cesaro et al., 2010). Macrophages and other immune cells express both pannexins and the P2X7Rs, in particular, PANX1 is highly expressed in human and mouse macrophages and has been shown to co-immunoprecipitate with the P2X7R protein (Pelegrin and Surprenant, 2006; Silverman et al., 2009). Thus in the context of inflammation signaling, Panx1 is a key part of a multi-protein signaling cascade that links the P2X7 receptor to the components of the inflammasome resulting in the eventual release of cytokines and cell death (Silverman et al., 2009). At the same time, high concentrations of ATP in the extracellular space may inhibit the Panx1 channel and thus Panx1 acts as a regulator of its own function through negative feedback inhibition (Figure 1B, black looped arrow) (Dubyak, 2009; Qiu and Dahl, 2009). It is possible that inhibition Panx1 function contributes to macrophage death or to conversion of pro-inflammatory M1 macrophages to pro-healing M2 cells that show a higher level of ATP and AMP hydrolysis (Zanin et al., 2012).

Perturbation studies strongly support the notion that Panx1 is important component of healing process and functions downstream of P2X7R (Wang et al., 2013). In particular, genetic ablation of P2X7R often results in decreased expression of Panx1 in correlation with delay in wound healing. For example, in P2X7-null corneas, Panx1 was absent from the wound edge and this was associated with delayed corneal re-epithelialization (Mayo et al., 2008). This suggests that P2X7R recruits Panx1 to mediate key inflammatory and regeneration processes.

Panx1 may also function through communicating with the cytoskeleton. Recent work demonstrated an interaction between Panx1 and both actin and actin-related protein 3 (ARP3), an actin cytoskeleton modulating protein (Bhalla-Gehi et al., 2010; Wicki-Stordeur et al., 2012). Moreover, Bao and co-authors suggest that ATP release by Panx1 channels initiates a signaling cascade that regulates actomyosin mediated cellular mechanics during cell migration (Bao et al., 2004, 2012). Thus, through modulating the cytoskeleton, Panx1 may control cell migration and/or cell process extension suggesting during wound healing.

An exciting emerging area is the role of pannexins in regulation of stem cell differentiation. Several new publications indicate that pannexins are expressed in various types of stem and progenitor cells and may be involved in regulation of their differentiation (Turmel et al., 2011; Cea et al., 2012; Wicki-Stordeur and Swayne, 2013). For example, extracellular ATP signaling influences myogenesis and regeneration of skeletal muscle (Martinello et al., 2011), and it is reported that ATP signaling during myoblast differentiation is mediated through Panx1 hemichannels (Cea et al., 2012).

Panx 1 is also found in multiple glandular tissues including sebaceous, pituitary, mammary, harderian, and lacrimal glands (neXtProt (human proteins) database, http://www.nextprot.org/db/entry/NX_Q96RD7/expression) (Li et al., 2011a; Cowan et al., 2012), however the role of Panx1 in these tissues is not well defined.

Panx2 and 3 were also proposed as regulators of cells differentiation/regeneration. In particular, Panx2 was implicated in regulation of neuronal cell differentiation. It has been shown that Panx2 protein is differentially expressed by multipotential neuronal progenitor cells and mature neurons. Stem-like neural progenitor cells express an S-palmitoylated intercellular localized Panx2 that prevents their differentiation, while committed differentiating neurons express mature (non-palmitoylated) Panx2 localizing at the plasma membrane. Moreover, knockdown of palmitoylated Panx2 significantly accelerated the rate of neuronal differentiation (Swayne et al., 2010). In the mammalian epidermis, Panx1 is co-expressed with Panx3, which plays a key role in keratinocyte differentiation (Celetti et al., 2010). Panx3, which is expressed in cartilage, has been reported to regulate chondrocyte proliferation and differentiation (Iwamoto et al., 2010). Panx3 also promotes differentiation of osteoblasts and *ex vivo* growth of metatarsals (Ishikawa et al., 2011). These studies suggest that Panx3 functions to switch the chondrocyte and osteoblasts cell fate from proliferation to differentiation, possibly through regulation of intracellular ATP/cAMP levels (Iwamoto et al., 2010; Penuela et al., 2012a), and may serve an important role in cartilage and bone development (Bond et al., 2011). Thus these studies point to key roles for pannexins in cell differentiation.

The overarching paradigm prompted by the numerous studies of pannexin function in cell culture, animal models, and clinically, is that Panx genes have a dual role in inflammation and tissue regeneration (Mohammad and Habib, 2014). Thus extracellular ATP signaling via purinergic receptors leading to formation of pannexin hemichannels, is a common pathway for inflammasome activation (Burnstock, 2006; Seminario-Vidal et al., 2009; Riteau et al., 2010, 2012; Hung et al., 2013; Lutz et al., 2013; Wang et al., 2013; Martins et al., 2014) and later for progenitor cell migration, proliferation, and differentiation during wound healing and tissue regeneration (Filbin, 2006; Mayo et al., 2008; Cea et al., 2012; Wicki-Stordeur and Swayne, 2013).

## Role of connexin hemichannels in inflammation and regeneration

Massive ATP release by macrophages and, as described more recently, by other cells (epithelial, endothelial, glial, neuronal), that activates the purinergic signaling cascade has been long implicated in inflammatory responses (Schenk et al., 2008; Silverman et al., 2009; Riteau et al., 2010; Gulbransen et al., 2012). Are the connexin hemichannels involved in this process? Connexins and pannexins present similar membrane topology, however, there is only 16% overall identity when their full-length amino acid sequences are compared (Orellana et al., 2012). Many cells/tissues express both pannexins and connexins. In addition to forming gap junction, connexins are also able to form active hemichannels in non-junctional membrane, that can release Ca^+^ and ATP (Bennett et al., 2003; Contreras et al., 2003). Similar to pannexins, opening of connexin hemichannels appears to be involved in many physiological and pathological cellular responses, including cell proliferation, inflammation, and cell death (Decrock et al., 2009; Garre et al., 2010). It has been reported that proinflammatory molecules may enhance ATP release via connexin hemichannels in Cx43-expressing cells (De Vuyst et al., 2007).

In cortical and spinal astrocytes, the activity of Cx43 hemichannels is strongly enhanced by application of proinflammatory molecules, such as tumor necrosis factor alpha (TNF-α), IL-1β, or fibroblast growth factor 1 (FGF-1) (Retamal et al., 2007; Garre et al., 2010). In spinal astrocytes, both pannexin (Panx1) and connexin (Cx43) hemichannels respond to FGF1 treatment by ATP secretion and by increased permeabilization to relatively large fluorescent tracers (ethidium and lucifer yellow) (Garre et al., 2010). However Panx1 hemichannels respond faster and have a leading role in ATP secretion and dye uptake (Garre et al., 2010). It has been proposed that FGF1/ATP-mediated activation of P2X7Rs, could lead to opening of Panx1 hemichannels and further release of ATP, and later significant accumulation of ATP, induces opening of Cx43 hemichannels, which also release ATP. Thus in both spinal and cortical astrocytes, cytokines also reduce dye coupling and the number of Cx43 gap junctions (Retamal et al., 2007; Garre et al., 2010). The mechanism of delayed opening of connexin hemichannels is not yet clear. It is possible that, in contrast to pannexins, opening of connexin hemichannels is regulated by a different, more complex or “slower” signaling pathway, or that connexin hemichannels need to be newly assembled in response to increasing concentration of ATP or pro-inflammatory signals.

## Conclusions

Pannexin hemichannels might serve as primary sensors of cell micro-environmental changes that allow cells within a tissue to respond proactively to environmental stresses, such as mechanical damage, microbial invasion, or metabolic stress, while connexin hemichannels may be critical to maintain this stress response. Due to wide expression in multiple tissues and involvement in numerous cellular functions, pannexins should be considered as potential therapeutic targets for diseases and conditions such as immune disorders, cancer, and acute inflammation, or for enhancement of progenitor cell proliferation, migration, and differentiation during wound healing and regeneration. However the precise role of pannexins in the delicate balance between release of pro-inflammatory molecules leading to cell death, and tissue regeneration needs to be further understood.

Defining the distinct roles of pannexin hemichannels in different physiological processes or at different stages of the physiological process such as cell/tissue regeneration provides the possibility for these channels and ATP release to be ultimately targeted in a context-dependent manner.

### Conflict of interest statement

The authors declare that the research was conducted in the absence of any commercial or financial relationships that could be construed as a potential conflict of interest.
